# AMPLIFY-NEOVAC: a randomized, 3-arm multicenter phase I trial to assess safety, tolerability and immunogenicity of IDH1-vac combined with an immune checkpoint inhibitor targeting programmed death-ligand 1 in isocitrate dehydrogenase 1 mutant gliomas

**DOI:** 10.1186/s42466-022-00184-x

**Published:** 2022-05-23

**Authors:** Lukas Bunse, Anne-Kathleen Rupp, Isabel Poschke, Theresa Bunse, Katharina Lindner, Antje Wick, Jens Blobner, Martin Misch, Ghazaleh Tabatabai, Martin Glas, Oliver Schnell, Jens Gempt, Monika Denk, Guido Reifenberger, Martin Bendszus, Patrick Wuchter, Joachim P Steinbach, Wolfgang Wick, Michael Platten

**Affiliations:** 1 DKTK (German Cancer Consortium) Clinical Cooperation Unit (CCU) Neuroimmunology and Brain Tumor Immunology, German Cancer Research Center (DKFZ), Heidelberg, Germany; 2 Department of Neurology, Medical Faculty Mannheim, MCTN, University of Heidelberg, Mannheim, Germany; 3 National Center for Tumor Diseases (NCT) Trial Center, NCT, Heidelberg, Germany; 4 Immune Monitoring Unit, NCT, Heidelberg, Germany; 5 Faculty of Biosciences, University Heidelberg, Heidelberg, Germany; 6 Neurology Clinic, Heidelberg University Hospital, University of Heidelberg, Heidelberg, Germany; 7 NCT, Heidelberg, Germany; 8 Department of Neurosurgery, Klinikum Grosshadern, Ludwig-Maximilians-University, Munich, Germany; 9 Department of Neurosurgery, Charité Medical Center, University of Berlin, Berlin, Germany; 10 Department of Neurology and Interdisciplinary Neuro-Oncology, University Hospital Tübingen, Hertie Institute for Clinical Brain Research, DKTK, DKFZ Partner Site, Eberhard Karls University Tübingen, Tübingen, Germany; 11 Division of Clinical Neurooncology, Department of Neurology and (DKTK) Partner Site, University Hospital Essen, University Duisburg-Essen, Essen, Germany; 12 Department of Neurosurgery, University Hospital Freiburg, Freiburg, Germany; 13 Department of Neurosurgery, Klinikum Rechts Der Isar, School of Medicine, Technical University Munich, Munich, Germany; 14 Institute of Cell Biology, Department of Immunology, University of Tübingen, Tübingen, Germany; 15 Institute of Neuropathology, Heinrich Heine University Düsseldorf, Medical Faculty, University Hospital Düsseldorf, Düsseldorf, Germany; 16 Department of Neuroradiology, Heidelberg University Hospital, Heidelberg, Germany; 17 Institute of Transfusion Medicine and Immunology, Medical Faculty Mannheim, Heidelberg University, German Red Cross Blood Service Baden-Württemberg - Hessen, Mannheim, Germany; 18 Frankfurt Cancer Institute (FCI), University Hospital Frankfurt, Goethe University, Frankfurt am Main, Germany; 19 DKTK CCU Neurooncology, DKFZ, Heidelberg, Germany

**Keywords:** Isocitrate dehydrogenase 1, Peptide vaccine, Astrocytoma, Oligodendroglioma, Recurrent glioma, Window-of-opportunity, Avelumab, T cell receptor sequencing, Brain tumors, Immune checkpoint inhibition

## Abstract

**Introduction:**

Isocitrate dehydrogenase (IDH) mutations are disease-defining mutations in IDH-mutant astrocytomas and IDH-mutant and 1p/19q-codeleted oligodendrogliomas. In more than 80% of these tumors, point mutations in IDH type 1 (IDH1) lead to expression of the tumor-specific protein IDH1R132H. IDH1R132H harbors a major histocompatibility complex class II (MHCII)-restricted neoantigen that was safely and successfully targeted in a first-in human clinical phase 1 trial evaluating an IDH1R132H 20-mer peptide vaccine (IDH1-vac) in newly diagnosed astrocytomas concomitant to standard of care (SOC).

**Methods:**

AMPLIFY-NEOVAC is a randomized, 3-arm, window-of-opportunity, multicenter national phase 1 trial to assess safety, tolerability and immunogenicity of IDH1-vac combined with avelumab (AVE), an immune checkpoint inhibitor (ICI) targeting programmed death-ligand 1 (PD-L1). The target population includes patients with resectable IDH1R132H-mutant recurrent astrocytoma or oligodendroglioma after SOC. Neoadjuvant and adjuvant immunotherapy will be administered to 48 evaluable patients. In arm 1, 12 patients will receive IDH1-vac; in arm 2, 12 patients will receive the combination of IDH1-vac and AVE, and in arm 3, 24 patients will receive AVE only. Until disease progression according to immunotherapy response assessment for neuro-oncology (iRANO) criteria, treatment will be administered over a period of maximum 43 weeks (primary treatment phase) followed by facultative maintenance treatment.

**Perspective:**

IDH1R132H 20-mer peptide is a shared clonal driver mutation-derived neoepitope in diffuse gliomas. IDH1-vac safely targets IDH1R132H in newly diagnosed astrocytomas. AMPLIFY-NEOVAC aims at (1) demonstrating safety of enhanced peripheral IDH1-vac-induced T cell responses by combined therapy with AVE compared to IDH1-vac only and (2) investigating intra-glioma abundance and phenotypes of IDH1-vac induced T cells in exploratory post-treatment tissue analyses. In an exploratory analysis, both will be correlated with clinical outcome.

***Trial registration*:**

NCT03893903.

## Introduction

The SOC treatment of patients with malignant gliomas is—independent of molecular markers—still confined to surgery, irradiation and alkylating chemotherapy as targeted therapies or immune checkpoint inhibitor monotherapy to date have failed to prove superiority over SOC in controlled trials [1]. At the same time, novel concepts in immunotherapy have evolved with the identification of potential (neo)epitopes and tumor-associated cell surface proteins, and neoadjuvant clinical trials investigating the efficacy of checkpoint inhibitors [2–4]. Despite patients frequently undergoing resection of recurrent tumors, patient selection criteria for innovative immunotherapy in glioma have been hampered by the lack of availability of post-treatment tumor tissue. Neoepitope-specific vaccines have gained considerable interest also in a challenging disease such as glioma. The oncogenic IDH1R132H protein, resulting from disease-defining IDH driver mutations in more than 80% of astrocytoma and oligodendroglioma WHO grade 2–4 cases [5], displays a neomorphic enzymatic activity that causes intratumoral accumulation of the oncometabolite R-2-hydroxyglutarate (R-2-HG) [6]. Accumulation of R-2-HG drives malignant transformation and immune escape by DNA and histone hypermethylation, metabolic reprogramming and systemic as well as direct and indirect local immunosuppression, respectively [7–11]. Conversely, IDH1R132H was previously identified to contain an immunogenic neoepitope [12–14].

A peptide vaccine targeting IDH1R132H (IDH1-vac), previously established in MHC-humanized mouse models [13], has been successfully tested in a phase I first-in-human multicenter clinical trial by the Neurooncology Working Group (NOA) of the German Cancer Society (NOA-16 trial) [12]. NOA-16 has enrolled 33 patients and was completed in 2017 with 32 patients treated. The primary endpoints were met by demonstrating safety and immunogenicity of IDH1-vac in combination with SOC (radiation, alkylating chemotherapy or the combination of both). Although NOA-16 was not conceptualized to demonstrate clinical efficacy, circumstantial evidence of biological activity of IDH1-vac was observed [12]: first, the rate of inflammatory pseudoprogressions (PsPD) in NOA-16 was higher compared to the frequency observed in a molecularly matched control cohort. PsPD is defined as an increase of tumor size on T2- Fluid-attenuated inversion recovery (FLAIR) magnetic resonance imaging (MRI) sequences and/or the novel appearance or enlargement of contrast-enhancing lesions followed by stabilization or regression on follow-up MRI up to three (RANO) to six months (iRANO) after initiation of SOC and/or immunotherapy, respectively [15, 16]; second, as potential predictive biomarker, MHC class II-restricted antigen presentation of the IDH1R132H-derived neoepitope visualized by proximity ligation assays [17] correlated with a compound variable of magnitude and persistence of peripheral IDH1-vac-induced T cell immune responses; Third, by using single cell sequencing technologies, the brain tumor homing of IDH1-vac-induced T cell clonotypes in an inflammatory PsPD following IDH1-vac treatment was demonstrated [18].

Immune checkpoint inhibitors (ICI) have failed to improve PFS or OS in unselected glioma patient cohorts with some evidence that neoadjuvant treatment is associated with intratumoral inflammatory reactions and favorable outcomes [3]. Avelumab (AVE) is a humanized anti- programmed death-ligand 1 (PD-L1) antibody approved for patients with Merkel cell carcinoma and urothelial cancer. Currently, 6 clinical trials investigating AVE in glioma patients have been registered, of which 2 are actively recruiting, including the AMPLIFY-NEOVAC trial (clinicaltrials.gov). The most recent data from clinical trials treating newly diagnosed (90% IDH-wildtype, NCT03047473) and recurrent glioblastoma patients (63% IDH-wildtype, NCT03291314) in combination with AVE demonstrated no beneficial OS nor PFS. Another AVE combination trial for glioblastoma patients administers VXM01, an investigational oral VEGFR-2 vaccine, to patients with progressive glioblastoma, and is currently ongoing (NCT03750071). For patients with IDH-mutant gliomas, AVE has been under investigation in one phase 2 single-arm trial for IDH-mutant secondary glioblastoma in combination with hypofractionated radiation, which has been completed in August 2019, yet results are awaited (NCT02968940).

## Methods

### Aim of the trial

AMPLIFY-NEOVAC will address safety and tolerability of the combination of IDH1-vac with AVE in patients with IDH1R132H-positive glioma and seeks to explore predictive biomarkers for response to ICI in post-treatment tumor tissues.

### Study description and study design

**Fig. 1:**
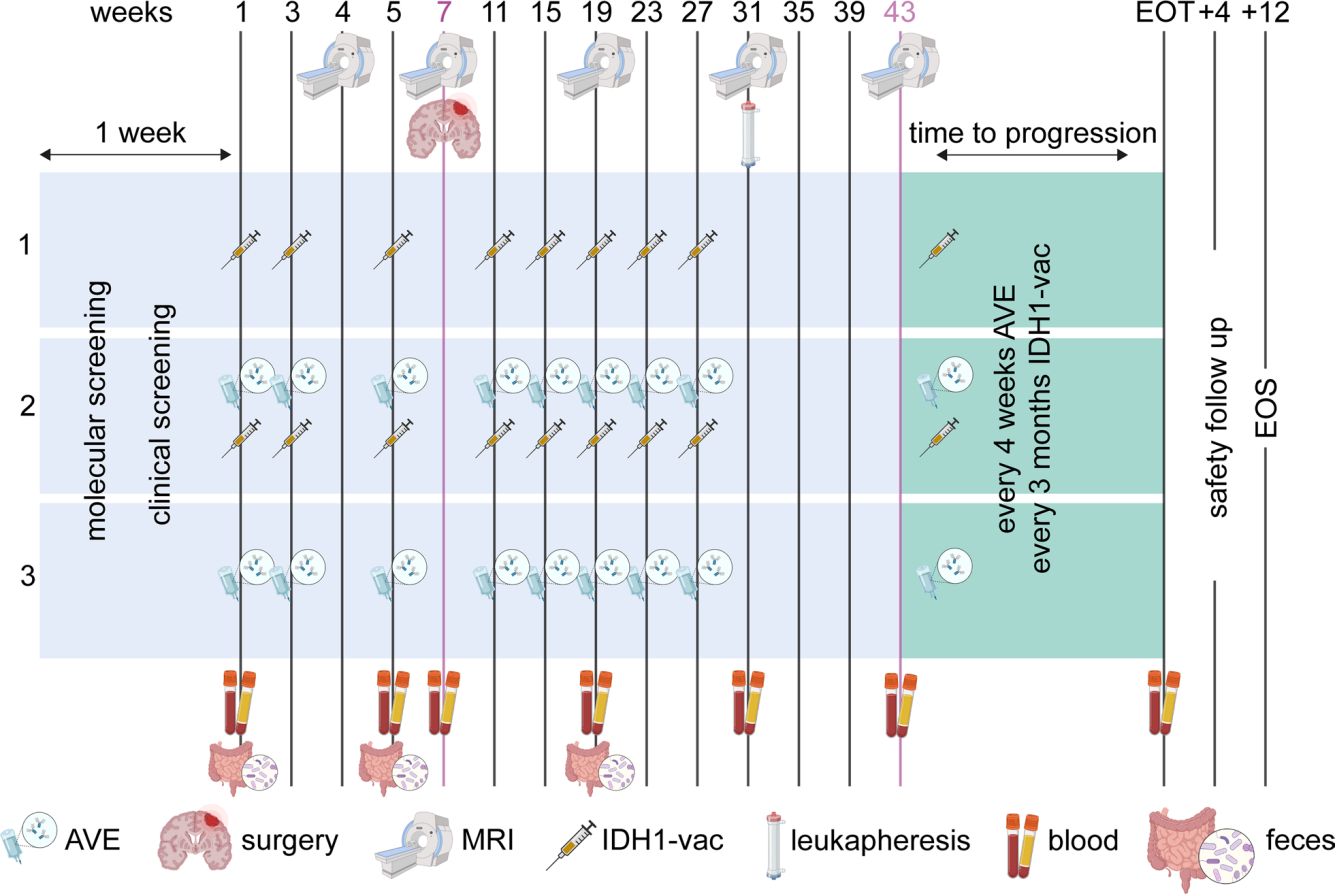
AMPLIFY-NEOVAC flow chart. Neoadjuvant and adjuvant immunotherapy will be administered to 48 evaluable patients. In arm 1, 12 patients will receive IDH1-vac; in arm 2, 12 patients will receive the combination of IDH1-vac and AVE, and in arm 3, 24 patients will receive AVE only. Until disease progression according to iRANO criteria, treatment will be administered over a period of maximum 43 weeks (primary treatment phase) followed by facultative maintenance treatment

**Fig. 2:**
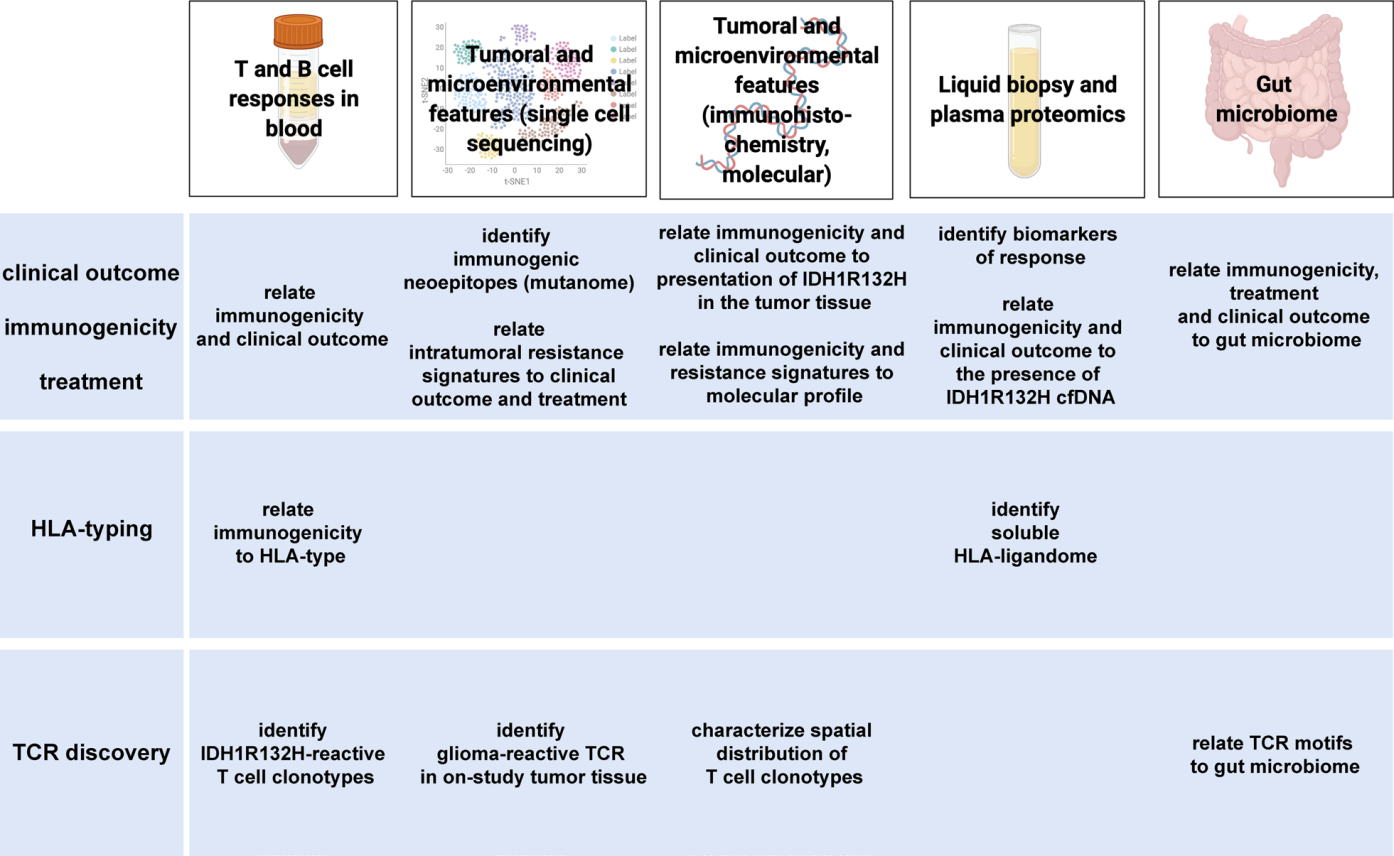
Summary of exploratory analyses within AMPLIFY-NEOVAC. T cell and B cell responses will be assessed as primary immunogenicity endpoints. Immunogenicity will be quantitatively and qualitatively correlated with supertype of human leukocyte antigens (HLA) and molecularly-defined IDH1R132H-specific T cells by single cell sequencing technologies. On-trial tumor tissues will be dissociated and TIL will be subjected to single cell sequencing to identify and characterize glioma-reactive T cells and treatment arm (1–3)-specific microenvironmental reprogramming. Liquid biopsies and fecal samples will be subjected to proteomic and transcriptomic analyses, respectively, and correlated to immunogenicity data and clinical endpoints

The open-label window-of-opportunity trial will enroll 48 evaluable patients with IDH1R132H-positive astrocytoma, CNS WHO grade 2, 3 or 4, or IDH1R132H-positive and 1/19q-codeleted oligodendroglioma, WHO grade 2 or 3 [5], progressive after radiotherapy and alkylating chemotherapy and eligible for re-resection. The trial treatment consists of a neoadjuvant and adjuvant mono-immunotherapy or combinatorial immunotherapy in three different arms. Study treatment will be offered at nine German trial sites within the German Cancer Consortium (DKTK) and the NOA. Treatment will continue until disease progression according to iRANO criteria [16]. The primary treatment phase is 43 weeks (last administration in week 39) followed by facultative maintenance treatment. Disease activity will be assessed by MRI every three months (Fig. 1). For primary endpoints and exploratory objectives, longitudinal blood sampling will be performed throughout the primary and maintenance treatment phases for exploration of research parameters (Fig. 2). A leukapheresis will be performed at visit 13 (week 31) for assessment of immunogenicity endpoints and exploratory objectives. In case of logistic difficulties, leukapheresis is replaced by collection of 200 ml heparin blood instead.

### Arms and interventions

After diagnosis of recurrent disease on imaging, patients will be randomized in a 1:1:2 ratio into three arms. 12 patients in arm 1 will receive three treatments with IDH1-vac in two-week intervals. IDH1-vac consists of a 20-mer IDH1R132H (p123-142) peptide emulsified in Montanide (ISA50) that is subcutaneously administered in combination with topical imiquimod, a toll-like receptor 7/8 (TLR7/8) agonist (5%, Aldara®) [12]. 12 patients in arm 2 will receive three administrations of IDH1-vac in combination with three doses of avelumab in two-week intervals. 24 patients in arm 3 will receive three doses of avelumab in two-week intervals. Although more recently it has been demonstrated that alkylating chemotherapy-associated mutations are subclonal [19], in arm 3, 24 patients will be treated with avelumab to probe the hypothesis that therapy-induced hypermutation (expected in 39–57% of cases as previously described in IDH-mutant astrocytomas and oligodendrogliomas after treatment with alkylating chemotherapy) [20] associated with an increased objective response rate. After 6 weeks of treatment (three neoadjuvant treatment time points), all patients (Arms 1–3) will undergo planned partial or complete re-resection. To ensure planned operability, a safety MRI will be performed three weeks after initiation of the study treatment. Four weeks after surgery, treatment will be resumed consisting of five additional IDH1-vac treatments (Arms 1 and 2) in 4-week intervals, followed by maintenance vaccines until progression in three months’ intervals after a treatment pause of 16 weeks at the end of the primary treatment phase. Avelumab will be administered in monthly intervals in Arms 2 and 3 starting four weeks after the surgery until progression. Progression will be determined according to iRANO.

### Outcome measures

Key outcome measures will be safety and immunogenicity of IDH1-vac (Arms 1 and 2) based on peripheral and intratumoral immune analyses assessed at the end of the primary treatment phase, i.e. 9 months after on-trial re-resection (week 43). In all study arms, additional exploratory analyses will determine efficacy, dependent on predictive molecular immune and imaging biomarkers, such as presentation of the IDH1R132H epitope within the pre-treatment tumor tissue, tumor microenvironmental molecular and transcriptomic profiles, and, in case of Arm 3 (AVE only), increased alkylating-therapy associated mutational load (Fig. 2). Immunogenicity will be assessed by the Central Immune Laboratory (CIL) following its standard operation procedures. Such immune monitoring will be based on peripheral blood mononuclear cell (PBMC) analyses, serum samples, and tumor-infiltrating T cell (TIL) cultures freshly isolated from glioma tissue collected within the study (week 7). PBMC and TIL samples will be analyzed for the occurrence of IDH1R132H-specific T cell responses using IFN-ɣ Enzyme-linked Immuno Spot (ELISpot) assay. Serum samples will be analyzed for IDH1R132H-specific antibodies using Enzyme-linked Immunosorbent Assay (ELISA). TIL cultures will be subjected to IDH1R132H- and glioma-reactive TCR identification and transcriptomic analyses using single cell RNA and VDJ sequencing.

For safety assessment, patients will be medically reviewed at each visit, including assessment of concomitant medications and adverse events (AE). All AEs will be graded according to National Cancer Institute Common Terminology Criteria for Adverse Events (CTCAE) version 5.0. Primary safety endpoint is the Regime Limiting Toxicity (RLT) until end of primary treatment phase (week 43, last IMP administration in week 39). RLT is defined as (1) any CTCAE grade 4 toxicity (except for single laboratory values out of normal range that are unlikely related to study treatment as assessed by the principal investigator, do not have any clinical correlate, and resolve within seven days with adequate medical management); (2) any injection site reaction of ≥ CTCAE grade 3 that persists after four weeks; (3) any other hypersensitivity, anaphylaxis or local allergic reaction ≥ CTCAE grade 3; (4) CTCAE grade 4 brain edema; (5) autoimmunity ≥ CTCAE grade 3; (6) change in Eastern Co-operative Oncology Group (ECOG) Performance Scale (PS) to ≥ 3 (Karnofsky Performance Index (KPI) to ≤ 40%) that does not resolve to ECOG-PS ≤ 2 (KPI ≥ 50%) within 14 days (infusions should not be given on the following cycle, if the ECOG PS-is ≥ 3 (KPI ≤ 40%) on the day of study drug administration); (7) CTCAE grade 3 toxicity to organs, but excluding the following: transient (≤ 6 h) CTCAE grade 3 flu-like symptoms or fever, which are controlled with medical management, transient (≤ 24 h) CTCAE grade 3 fatigue, local reactions, headache, nausea, emesis that resolves to CTCAE grade ≤ 1, single laboratory values out of normal range (excluding CTCAE grade ≥ 3 liver function test increase) that are unlikely related to study treatment according to the principal investigator, do not have any clinical correlate, and resolve to CTCAE grade ≤ 1 within 7 days with adequate medical management, tumor flare phenomenon defined as local pain, irritation, or rash localized at sites of known or suspected tumor; and (8) death (including death due to disease progression). Adverse Events of Special Interest are (1) exposure during pregnancy, (2) exposure during lactation, (3) death due to disease progression and (4) occupational exposure.

Secondary objectives of the study, among others, include (1) assessing immunogenicity of IDH1-vac alone or in combination with AVE, (2) assessing efficacy of AVE in patients with treatment-induced hypermutator phenotype tumors compared to non-hypermutator phenotype tumors, (3) assessing overall survival (OS), progression-free survival (PFS), and overall response rate (ORR), and (4) associative studies on immunogenicity and clinical outcome parameters. ORR is defined by the rate of patients with complete or partial response or stable disease at the end of the primary treatment phase according to iRANO by central independent review of MRI [16].

### Eligibility criteria

Main patient inclusion criteria are as follows: age ≥ 18 years; first, second or third recurrence of a histologically confirmed IDH1R132H-positive 1p/19q-codeleted oligodendroglioma, CNS WHO grade 2 or 3, or astrocytoma, CNS WHO grade 2, 3 or 4, progressive after radiotherapy and alkylating chemotherapy eligible for complete or partial re-resection and the re-resection must be postponable for seven weeks; availability of FFPE tissue from previous resection (biopsy sufficient); patients have received radiotherapy (54–60 Gy) and alkylating chemotherapy; patients are at least three months off radiotherapy; patients should not require > 2 mg/day dexamethasone (or equivalent), and patients´ KPI must be ≥ 70.

### Contacts

Sponsor: German Cancer Research Center, Im Neuenheimer Feld 280, 69120 Heidelberg.

Investigators: Michael Platten, Neurology Clinic, Medical Faculty Mannheim, University Heidelberg; Wolfgang Wick, Neurology Clinic, Medical Faculty Heidelberg, University Heidelberg; Joachim Steinbach, Dr. Senckenberg Institute of Neurooncology, Frankfurt, Germany.

## Perspective

IDH mutations are disease-defining mutations in oligodendrogliomas and astrocytomas. Most frequently, they result in the generation of a mutant protein with a neomorphic enzymatic activity, namely excessive R-2-HG production. Oncogenic R-2-HG accumulation has been the scientific rationale to develop specific inhibitors of mutant IDH, which are capable of suppressing tumor growth in preclinical cancer models and early clinical trials. In addition, IDH1R132H harbors a tumor-specific neoepitope with high uniformity and penetrance, that is expressed in all tumor cells, and typically preserved in recurrent tumors. IDH1-vac safely targets IDH1R132H in newly diagnosed astrocytomas with transient or sustained immune responses in 93.3% of treated patients [12]. The successor trial, AMPLIFY-NEOVAC, specifically aims at demonstrating safety of enhanced peripheral IDH1-vac-induced T cell responses by combined therapy with AVE and investigating intra-glioma abundance and phenotypes of IDH1-vac- and AVE-induced T cells in exploratory post-treatment tissue analyses. Moreover, in the framework of the Collaborative Research Center 1389 (https://www.unite-glioblastoma.de), it will comprehensively assess intratumoral, microbiome-associated, proteomic and peripheral cellular determinants of response and resistance to peptide vaccine and/or ICI in patients with IDH1-mutated gliomas in an unprecedented manner. To our knowledge, this is the first ICI clinical trial in patients with IDH1R132H-positive gliomas which applies ICI in a neoadjuvant setting. While closely monitoring safety, the window-of-opportunity design allows for systematic molecular and immunological analysis of post-treatment tumor tissue with a comprehensive predefined standardized profiling in great and unprecedented detail. At the same time, neoadjuvant immunotherapy in this trial may be more effective, as suggested by trials using neoadjuvant ICI in recurrent glioblastoma [3].

## Data Availability

Not applicable.

## Abbreviations

AE: Adverse events
AVE: Avelumab
CIL: Central Immune Laboratory
CTCAE: Common Terminology Criteria for Adverse Events
DKTK: German Cancer Consortium
ECOG-PS: Eastern Co-operative Oncology Group (ECOG) Performance Scale (PS)
ELISA: Enzyme-linked Immunosorbent Assay
ELISpot: Enzyme-linked Immuno Spot
FLAIR: Fluid-attenuated inversion recovery
ICI: Immune checkpoint inhibitor
IDH1R132H: Isocitrate dehydrogenase 1, amino acid exchange R132H
IDH1-vac: IDH1R132H targeting 20-mer peptide vaccine
IMP: Investigational medicinal product
iRANO: Immunotherapy response assessment for neuro-oncology
KPI: Karnofsky Performance Index
MHCII: Major histocompatibility complex class II
MRI: Magnetic resonance imaging
NCI: National Cancer Institute
NOA: Neurooncology Working Group of the German Cancer Society
ORR: Overall response rate
OS: Overall survival rate
PBMC: Peripheral blood mononuclear cell
PD-L1: Programmed death-ligand 1
PFS: Progression free survival rate
R-2-HG: R-2-hydroxyglutarate
RLT: Regime Limiting Toxicity
SOC: Standard of care
TIL: Tumor-infiltrating T cell
